# Mixed Reality in Clinical Settings for Pediatric Patients and Their Families: A Literature Review

**DOI:** 10.3390/ijerph21091185

**Published:** 2024-09-05

**Authors:** Jae Eun Sin, Ah Rim Kim

**Affiliations:** Department of Nursing, College of Healthcare Science, Far East University, Eumseong-gun, Gamgok-myeon 27601, Republic of Korea

**Keywords:** augmented reality, mixed reality, systematic review, review, pediatric patient, intervention, education, clinical setting

## Abstract

In the post-pandemic context, there has been an increasing demand for technology-based interventions in education and healthcare systems, such as augmented and mixed reality technologies. Despite the promising outcomes of applying mixed reality (MR), there is limited aggregated evidence focusing on child–patient interventions in hospital-based or clinical settings. This literature review aimed to identify and synthesize existing knowledge on MR technologies applied to pediatric patients in healthcare settings. Following the Preferred Reporting Items for Systematic Reviews and Meta-Analyses (PRISMA) guidelines, a comprehensive search of the Scopus and Web of Science databases was conducted to identify articles published in the last 10 years that address the application of augmented and/or MR technologies in pediatric hospital settings or clinical environments to improve patient and family outcomes. A total of 45 articles were identified, and following a rigorous screening and eligibility process, 4 review articles were selected for qualitative synthesis. From these reviews, 10 studies with relevant interventions and measured effects were extracted. The extracted studies were analyzed based on eight key attributes: country of origin, study design, characteristics of the study population, primary clinical setting, type of MR device used, nature of the intervention, variables measured, and significant effects observed in the outcome variables. The analysis revealed diverse approaches across different clinical settings, with a common focus on improving both emotional well-being and learning outcomes in pediatric patients and their families. These findings suggest that MR-based pediatric interventions generally provide children and their parents with positive emotional experiences, enhancing both learning and treatment outcomes. However, the studies reviewed were heterogeneous and varied significantly in terms of clinical settings and MR applications. Future research should focus on developing more controlled study designs that specifically target the pediatric population to strengthen the evidence base for MR interventions in healthcare.

## 1. Introduction

### 1.1. Importance of New Technologies in Health and Education

The coronavirus disease 2019 pandemic and the emergence of the new normal have underscored the importance of new technologies in health and education for better outcomes, such as augmented reality (AR) and mixed reality (MR) [1,2]. These technologies are increasingly recognized as vital tools in modern healthcare and educational systems.

### 1.2. Definitions and Distinctions between AR, VR, and MR

AR is defined as a display-based system involving a combination of physical and virtual three-dimensional (3D) imagery, interacting within a real-world environment [3,4]. In virtual reality (VR) systems, users are immersed in a computer-generated virtual world, typically using a headset to block out visual stimuli from the real environment. AR technology has addressed the limitation of VR, which is a lack of a relationship with real space, to create an augmented world that enables users to view the real world by overlaying virtual elements or incorporating virtual information into the user’s real surroundings [3,5,6]. In contrast, the MR environment merges the real and virtual worlds through a window created between them by combining and mapping real-world and virtual objects, which leads users to interact in real-time [6]. Extended Reality (XR or xReality; i.e., metaverse) technologies is an umbrella term that covers all forms of new realities (e.g., VR, AR, MR, and various subforms) [7,8].

### 1.3. Conceptual Challenges and Frameworks

**Figure 1:**
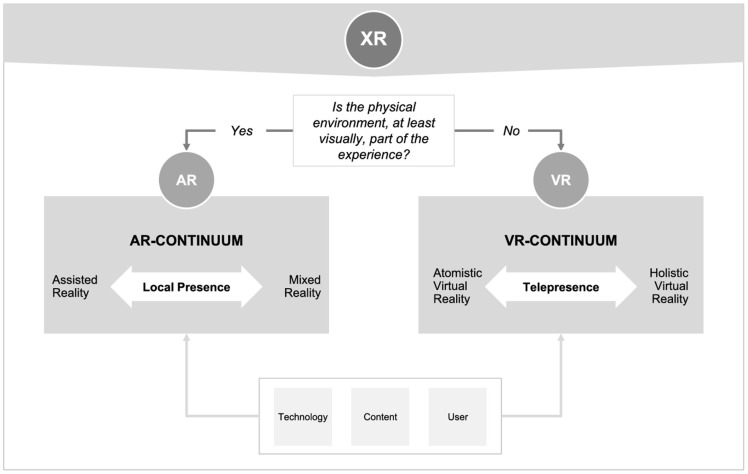
XReality (XR) framework: augmented and virtual reality. Reproduced from Rauschnabel et al. (2022) [7].

No universally accepted single definition of MR exists, and its understanding depends on one’s context [9]. Additionally, the use of these terms among professionals and academics has been inconsistent because of the ambiguity and confusion around terms and concepts, such as AR and MR, in the literature [7]. However, AR and VR should be separated since they fundamentally differ in terms of user experience: AR and VR are based on local presence and telepresence-continuum, respectively [7,10]. This distinction is clearly illustrated in the XReality (XR) framework, which positions AR and VR along different continua based on the degree of user immersion and interaction with the physical environment (see Figure 1). Many consider MR synonymous with AR due to their shared characteristics in integrating digital content with the real world. However, MR extends beyond AR by allowing interaction between the digital and physical elements, creating a more immersive experience [9]. For example, studies by Solbiati et al. (2020) [10] and Gupta et al. (2022) [11] demonstrated how MR and AR can be perceived differently depending on the technological implementation and application context [12].

### 1.4. Technological Advancements and Applications

The fields of MR have been rapidly growing over the past decade; however, despite recent advancements highlighted in recent umbrella reviews, the application of MR technology in medical education still remains in its early stages, with limited substantial evidence to support its widespread adoption [13]. Regarding psychosocial benefits, the use of VR or AR devices can improve learning motivations and self-confidence through an immersive environment and interactive teaching strategy [14]. Additionally, VR may foster engagement and autonomous learning attitudes by providing psychological safety and enjoyable feelings in potential gamification settings [15]. Despite the advantages of VR, challenges such as reduced face-to-face communications, cost concerns, user attitudes, safety considerations, and VR-related side effects have been documented in its application in medical education and treatment settings, as highlighted in recent studies [16].

A comprehensive range of hardware and software technologies such as display, object recognition and tracking, image rendering, and interaction are prerequisites for developing VR or AR education and training systems with a high sense of immersion [17]. Although display technology constitutes the basis for VR and AR systems to deliver visual information, some display-related technical issues have remained challenging (e.g., high cost, insufficient viewing angle and resolution, image processing, and output latency) [17]. Nevertheless, simulation education is poised for continued expansion as technological advancements progress. Improved use of hand and voice controls and haptics will become feasible, and artificial intelligence (AI) technology will be integrated into real-life scenarios to greatly enhance learner interactions [15]. AR is well-suited for use in medical and nursing learning contexts because students do not require any material or resources beyond the devices and an internet connection [18]. Additionally, users can interact with each other using different devices, such as smart glasses-type products that are based on optical perspective technology [19]. Xu, Mangina, and Campbell [14] revealed that 11 of the 15 randomized control trials (RCTs) using VR HMD as interventions used Oculus Rift, HTC VIVE, Gear VR, and customized HMD, whereas the remaining studies used HoloLens and AiRScouter glasses as AR devices.

### 1.5. The Role of AR and MR in Modern Healthcare and Education

The third generation of ubiquitous computing and network environments has brought about a paradigm shift in education and healthcare systems. Digital health services based on information and communication technology, blockchains, digital twinning, and metaverses—such as AR, VR, MR, and robotics—greatly impact the interaction between patients and physicians [20]. Particularly, the release of consumer-grade VR and AR technologies leading to innovative learning can foster users’ hands-on learning experiences [21]. AR is well-suited for developing educational programs in the medical and manufacturing fields, where practical education is difficult to experience in the real world or is accompanied by high costs and risks [22]. Furthermore, AR technology, which has the advantage of educational affordance, allows learners to have a sense of reality and presence and independently construct knowledge [20]. While VR provides a completely virtual environment that allows learners to experience a sense of dissimilarity with reality, it can also enhance concentration by fully immersing users and minimizing external distractions. In contrast, AR-based learning detects real-time changes and facilitates information searches in real space, leading to significant learning effects [23,24]. Therefore, AR is an effective digital learning tool in medical education because it enables educators to design rich educational curricula and provide learners with an engaging learning experience [25]. Additionally, integrating AR, VR, and digital twins into healthcare can greatly enhance patient care. For example, AR can be used to create interactive educational tools that help patients understand their conditions and treatments. VR can provide immersive therapy environments for pain management and mental health support. Digital twins can simulate individual health data to personalize treatment plans. These technologies not only improve patient education and engagement but also contribute to more precise and effective healthcare, leading to better health outcomes and increased patient satisfaction.

### 1.6. Psychological and Physical Risks of AR and VR

While AR and VR offer significant potential benefits for education and training, they also present notable psychological and physical risks. Similar to other forms of media, these technologies can impose various stresses and discomforts on users. AR and VR environments can increase the cognitive load and psychological stress. Prolonged use may lead to mental fatigue and anxiety, particularly when users are exposed to distressing or unsettling content. For instance, the immersive nature of VR can amplify the impact of disturbing scenarios, potentially causing emotional discomfort [8]. Similarly, AR applications can introduce unsettling virtual objects or environments, intensifying psychological stress. The blending of virtual elements with the real world may create confusing experiences, contributing to anxiety and discomfort [26].

Physically, AR and VR can pose various issues due to the intense visual and sensory stimuli they deliver. VR environments are known to induce motion sickness and visual discomfort. Users may experience nausea, headaches, or even seizures from prolonged exposure to dynamic and immersive content [25]. Additionally, AR applications can lead to sensory overload, resulting in physical discomfort or strain. Research indicates that the sensory demands of AR can overwhelm users, causing physical stress and discomfort [27]. These risks highlight the necessity for effective safety measures and usage guidelines to protect users from adverse effects. Both AR and VR technologies require careful consideration of these risks in their design and application. Implementing robust safety protocols and conducting ongoing research are crucial for mitigating these risks and enhancing user experience. Addressing these concerns will help maximize the benefits of AR and VR while safeguarding users’ well-being.

### 1.7. Gaps in Research and the Need for Focused MR Interventions

However, in existing systematic reviews, the effectiveness of MR interventions for children, adolescents, and families was constrained by the diverse age groups and environmental settings. This study is crucial for selectively extracting and synthesizing intervention studies focused on pediatric patients in clinical settings, thereby overcoming this limitation. It underscores the significance of viewing MR interventions as non-pharmacological alternatives for children and adolescents who are vulnerable to pain and drug side effects in clinical settings, compared to medication or invasive treatments. Therefore, we conducted a literature review, also known as a meta-review, which provides a timely resource for decision-makers in healthcare systems [28] by synthesizing existing literature reviews and contributing to a more comprehensive understanding of the evidence than individual studies or reviews alone.

Our research question was, “Which interventions using MR technologies for patients and their families in pediatric clinical settings have a measured impact on patient and family health outcomes?” This question was defined according to a priori inclusion and exclusion criteria using the population/participants, intervention, comparison/context, outcome, and study design (PICOs) [28] (Table 1). This study aimed to (1) identify and synthesize existing review-level evidence on MR-based interventions for children and their families in hospital-based or clinical settings, (2) examine the general characteristics of the populations and interventions, (3) discuss the conceptualization of MR interventions and health outcomes, and (4) suggest potential gaps in the established knowledge and directions for future studies.

## 2. Materials and Methods

**Figure 2:**
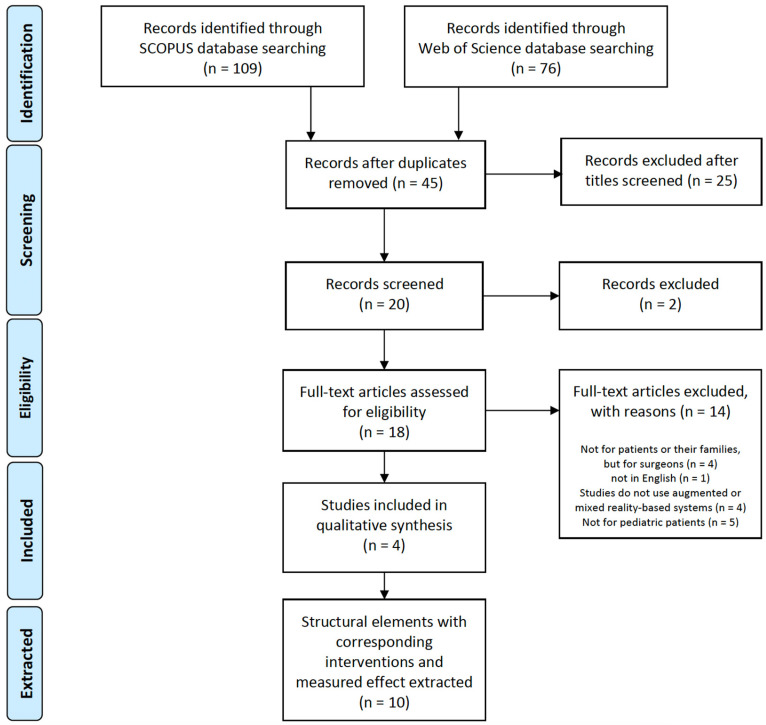
Study selection flow diagram.

This literature review adhered to the Preferred Reporting Items for Systematic Reviews and Meta-Analyses guidelines [29] and was conducted to consolidate the current knowledge on how MR technologies apply to and impact pediatric patients’ health outcomes and/or their family-related outcomes in clinical settings (Figure 2).

### 2.1. Identification

A search was performed in August 2022 to identify articles for inclusion in this review using the following two databases that index peer-reviewed articles: Scopus and Web of Science databases. The scope was defined as using AR and/or MR technologies in clinical services (settings) for pediatric patients and/or their families to improve patient and family-related outcomes. Table 2 presents the search queries and their results.

### 2.2. Screening and Eligibility

Articles were included if they were systematic reviews or meta-analyses focusing on interventions involving MR technologies in pediatric healthcare settings. After removing duplicates, 45 articles remained (Figure 2). An initial pre-screening process excluded 25 records based on titles and abstracts, as they did not meet the basic inclusion criteria, such as not being systematic reviews or meta-analyses or not including pediatric healthcare settings. This left 20 articles for further screening. During the screening phase, two additional articles were discarded because their target population was not patients or their families, but rather medical residents and students. To align with the review objectives, only participants who were pediatric patients and their families in clinical or hospital-based settings qualified for inclusion. Additionally, review articles published in English after 2012 were included, while those with a limited intervention focus, such as articles exclusively about VR technology or those involving only clinicians or surgeons as users, were excluded. The remaining 18 full-text articles were then assessed for eligibility. Importantly, systematic reviews that focused on the general population were included if they also contained studies involving pediatric patients. In such cases, only the data specific to pediatric patients and their families were extracted and analyzed for our review. This process led to the inclusion of 4 reviews in the qualitative synthesis, with a total of 10 studies extracted for detailed analysis.

### 2.3. Included Articles

Two reviewers independently screened all identified reviews, separately verified the correct exclusion of studies, and assessed the eligibility of the remaining articles. A third reviewer was consulted in cases where agreements were not reached. We conducted data extraction on the following two levels: first, our research yielded four review articles that contained MR interventions for children and their families in clinical services or hospital-based settings, and these articles were selected for final data extraction. Second, the four reviews were left as primary studies to extract the 10 studies with the corresponding interventions and measured effects. Finally, eligible articles were processed, and all interventions that affected the patients and/or their parents were extracted. We opted to only narratively synthesize the data due to the heterogeneity and the infeasibility of conducting a meta-analysis.

## 3. Results

### 3.1. Overview of Included Studies

#### 3.1.1. Search Results

Table 3 presents the domains of the four included systematic reviews [30,31,32,33], comprising technologies used for the intervention, main users, number of papers per review, method of analysis, study settings, and implications. Three of the included reviews used qualitative techniques, and one conducted quantitative synthesis. The included reviews were published in English between 2021 and 2022. Additionally, three reviews [31,32,33] focused on VR and AR technology-based interventions, whereas the other [30] included only AR-based patient education. The AR technology’s main users (target population) were patients with diverse conditions (chronic disease, pain, anxiety, autism spectrum disorder [ASD], and burn injuries) across clinical settings (patient education setting, operating room and recovery area, outpatient department, or burn care center). Furthermore, AR as a nonpharmacological intervention significantly affected patient-related outcomes [31,32,33], particularly in efficacy among patients with ASD. However, because of the limitations in study design and inadequate relevant evidence related to AR-based interventions, most reviews could not verify component effectiveness for pediatric patients by setting. Therefore, most reviews advocated for more high-quality trials.

#### 3.1.2. Study Characteristics

Ten findings were extracted from the four included reviews (Table 4) and grouped into the following eight characteristics of MR-based interventions: country, study design, population characteristics, primary clinical setting, MR device, intervention, variable, and significant effect of outcome variables. Of the 10 extracted studies, 3 were conducted in the United States of America, 2 in the United Kingdom, and the remaining in Spain, Taiwan, Indonesia, Colombia, and Australia.

### 3.2. General Characteristics of the Studies

#### 3.2.1. Methods

The extracted studies included four controlled trials (n = 4) and six uncontrolled trials (n = 6). Among the controlled trials, randomization was applied in three studies [34,36,43], while the fourth employed a mixed methods design that combined quantitative non-randomized controlled trials (non-RCTs) and qualitative semi-structured interviews [35]. Except for one case report [37], most uncontrolled design studies (quasi-experimental research design) had before-and-after comparisons within the intervention group [38,39,40,41,42]. Furthermore, the studies on ASD applied a within-subject experiment [38] or a single-subject experiment using the applied behavior analysis [41] or antecedent behavior and consequence method [39,40,42]. All selected studies were published in English.

#### 3.2.2. Study Population

In the 10 papers, 330 participants were included, most of whom were children and adolescents, although one study recruited young adults with ASD [42]. Although two studies examined the experiences of patients visiting a hospital without specifying their medical condition [34,35], most studies focused on patients with specific diseases and/or disorders or in clinical settings, such as having diabetes mellitus [36], acute burns [43], ASD [37,38,39,40,41,42], or waiting for upper gastrointestinal endoscopy [37]. However, an imbalance in sex distribution was observed in each study. Table 4 presents the number of participants included and the average or range of patient ages.

### 3.3. Description of the Interventions

#### 3.3.1. Clinical Settings of the MR Application

The included papers, while applying their MR-based intervention, denoted clinical settings as outpatient facilities or departments [34,43], outpatient and inpatient departments for a planned clinical procedure without moderate or severe cognitive impairment or a referral to psychological services for procedural anxiety [35], operating rooms during the induction of general anesthesia [37], or neurorehabilitation clinics [41]. However, the clinical environment where the intervention was applied was not described in detail in half the studies. Only one study provided information on participants as “conference attendees for patients with diabetes and relatives in 2016” rather than on specified intervention settings [36]. Some studies presented limited explanations of the intervention environment, such as a day treatment room [39], or participants’ recruitment settings, such as criteria for school inclusion [40]. Furthermore, two other studies did not reveal their clinical settings in detail [38,42]; therefore, we assumed that they were not hospitals but treatment settings.

#### 3.3.2. MR Devices and Software

Screen-based smart devices, such as smartphones or tablet personal computers (PCs), have been used to implement MR interventions [34,35,36,37,39,40,41]. Two studies did not use smart devices and instead used hand-held AR screens [43] or mirror AR display devices [38]. Only one study used smart glasses (Google Glass), which did not require additional physical hand-held screen devices, for the intervention [42].

Three MR development frameworks emerged from the 10 studies examined: Vuforia [34,36,39,41], ARKit [35], and ALVAR [38]. These frameworks were used in AR application systems across six studies. Additionally, one study employed an AR device with a 7-inch LCD screen connected to an Intel Pentium system [43]. However, other studies that employed wearable devices—such as headsets [37], smart glasses [42], the AR Picture Exchange Communication System device [40], and hand-held devices—did not disclose information about their software development kits (SDK). This highlights a gap in the reporting of the technological infrastructure supporting these MR interventions.

Vuforia, ARKit, and ALVAR were found in a recent exploration of the user interface and development frameworks on mobile AR to support the utilization of Unity [44]. This integrated tool is instrumental in generating interactive content, including 3D animations, architectural visualizations, real-time animations, and interactive game simulations. The tablet PC models used in the studies were limited to one of the following operating systems (OSs): iOS, Android OS, or Windows 8. Although ARKit operates exclusively on iOS platforms, both ALVAR and Vuforia demonstrate versatility by being compatible with Android OS, iOS, and Windows OS.

#### 3.3.3. MR-Based Interventions

(1) Overview of MR-Based Interventions

**Figure 3:**
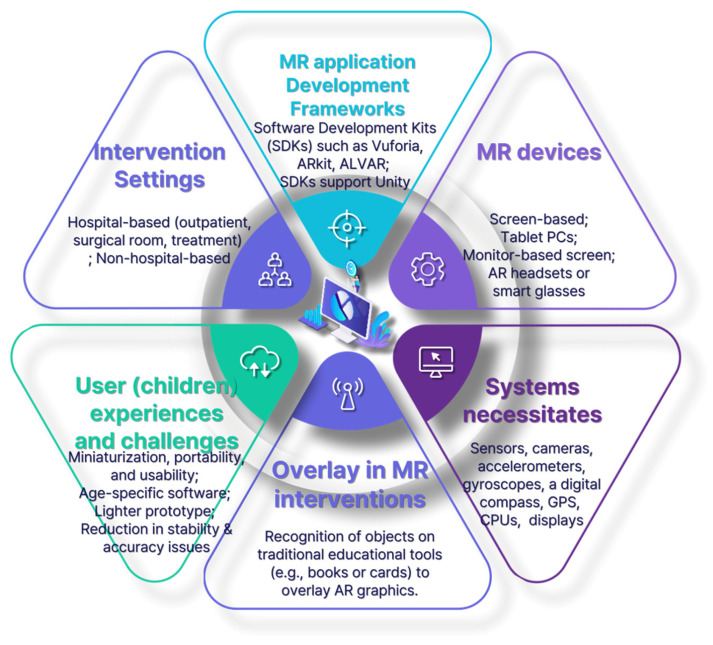
A framework of MR in clinical settings for pediatric patients and their families. AR, augmented reality; MR, mixed reality; CPU, central processing unit; GPS, Global Positioning System; PC, personal computer.

Figure 3 shows the results of this review, which is a diagram based on the MR framework. MR-based interventions were primarily conducted in environments where AR graphics generated by recognizing objects on traditional educational tools, such as books or cards, were overlaid to provide visual and auditory stimuli. Compared to VR, the AR system characterizes real and virtual content combinations and requires sensors, cameras, accelerometers, gyroscopes, a digital compass, a Global Positioning System, central processing units, and displays. Appropriate devices for AR include mobile devices, such as smartphones/tablets, head-up displays (HUD), smart glasses, smart lenses, or virtual retinal displays (VRD) [45]. For pediatric clinical purposes, ten studies included the use of mobile devices and smart glasses rather than HUDs, smart lenses, or VRDs. This preference could arguably be indicative of the ongoing trend toward miniaturization in AR devices, emphasizing enhanced portability and usability.

(2) MR Interventions in Hospital Settings

The intervention settings, spanning from 2008 to 2022, varied from hospital-based settings, such as outpatient clinics, surgical rooms, and treatment rooms, to non-hospital settings. Over the last 15 years, technological advancements and user experience enhancements have occurred:

- Early Adoption in Pain Management: Mott et al. [43], in 2008, represented the early adoption of MR in pediatric care. A child could visualize a 3D animation character from multiple angles in an MR system with audio narration, which made the child perform tasks. This highlights MR’s application in pain management and its ability to improve patient experiences in medical settings.
- Developmental and Educational Tools: By 2015, Bai et al. [38] established a setting for patients playing with augmented toys in a mirror MR display to improve and learn how to pretend to play, a crucial skill for autistic children. This showed the application of AR as a tool for social and cognitive development in children with ASD, promoting interaction and engagement. Similarly, a study by Calle-Bustos et al. [36] revealed MR’s role in interactive education for chronic health conditions, such as diabetes mellitus. An Android device overlaying MR food on a real dish was used in an AR game to support therapeutic education for children with diabetes mellitus, significantly contributing to self-management education in pediatric diabetes.
- Anxiety Reduction and Advanced MR Applications: In 2020, studies by Tait et al. [34] and Bray et al. [35] exemplified the maturity of MR technologies. Tait et al. used a printed storybook and an MR-enabled iPad program overlaying MR graphics, animations, and a chatbot with embedded interactive quizzes for information evaluation. Bray et al. employed the preloaded iPad Xploro^®^, a digital therapeutic (DTx) platform that adopts AR, gameplay, and artificial intelligence, providing information on health environments, key health staff, and hospital equipment. Another study by Libaw et al. [37] in 2020 furthered the application of MR in clinical settings by applying the AR “Jenny the Robot” distraction technique during mask induction to encourage patients to take deep breaths.

(3) MR Interventions in Non-Hospital Settings

Non-hospital settings, which were not described in detail in the literature, have mostly been used in studies targeting children with ASD:

- Social and Communication Skills Enhancement: Extracted from the primary study of Karami et al. [32], five individual studies on ASD conducted between 2015 and 2018 applied MR interventions for various social and communication skill-enhancing purposes. In a study by Chen et al. [39], AR-based Video-Modeling with Storybook (ARVMS)—comprising seven sessions—was devised to learn the facial expressions and emotions of others in social situations.
- Integration with Therapeutic Methods: Other studies targeting children with ASD, such as those by Kurniawan [40] and Nubia et al. [41], showed a continued trend toward using MR for enhancing communication and social skills. Kurniawan developed the Picture Exchange Communication System with AR-based multimedia using visual aids in a more interactive format to improve the communication abilities of children with ASD. In contrast, Nubia et al. introduced the process and simulation of an MR-based pictogram recognition task to improve the attention process and the appearance of verbal language in participants with ASD.
- Innovative Smart Glasses Applications: Vahabzadeh et al. [42] illustrated the innovative use of smart glasses-based interventions. Specifically, they reported that Empowered Brain, a smart glasses-based social communication and behavioral intervention, was used to improve the duration of gaze at faces and reduce ADHD symptoms in children, adolescents, and young adults with ASD.

### 3.4. Outcomes

#### 3.4.1. Variables

The outcome variables in the included studies from Urlings et al. [30] primarily focused on the intervention participants’ knowledge acquisition, satisfaction with AR use, and usability [34,35,36]. Tait et al. [34] implemented AR intervention focused on perceived knowledge, procedural anxiety, procedural satisfaction, procedural involvement, usability, and likability and found an effect of patient knowledge on the understanding of clinical research and perceptions of information delivery for children and parents [35], whereas Calle-Bustos et al. [36] focused on knowledge and usability.

AR programs in the operation setting [37] and dressing changes for patients with burns [43] were applied to discover the psychological effects of the medical experience. Libaw et al. [37] did not measure anxiety, although patients and their parents were interviewed about their AR experiences regarding anxiety and satisfaction after recovery in the post-anesthesia care unit. In contrast, Mott et al. [43] designated pain score, pulse rate, respiratory rate, oxygen saturation, and parental pain assessment scores as the main outcome variables.

In the studies focused on ASD, Bai et al. [38] employed a play observation scale using video analysis to examine the frequency and duration of pretend and constructive play, respectively; Chen et al. [39] used the instructor’s assessment of task performance, and Kurniawan [40] adopted the teacher’s evaluation of communication ability score. In the study by Nubia et al. [41], the number of children who completed the attention task, making sounds or movements, was measured in the appearance of verbal language and the children’s responsiveness to the therapists during interactions. Furthermore, Vahabzadeh et al. [42] used the caregiver-reported ADHD symptoms with hyperactivity subscale of the Aberrant Behavioral Checklist score.

#### 3.4.2. Main Effect

The three studies extracted from Urlings et al. [30] focused on knowledge gain, satisfaction, or usability as the major effects of MR-based interventions. Both the AR and control groups demonstrated significant improvements in post-test understanding without differences between the groups; however, some younger children perceived the amount of information in the AR program to be relatively extensive [34]. A study reported participants’ self-perceived knowledge gain, favoring AR and indicating a positive effect of AR on the perception of procedural knowledge [35]. Although the results were not statistically significant in this study, positive results were found for patient satisfaction and related outcomes, such as patient involvement and anxiety. Another study reported significant improvements and gains in knowledge between pre–post test results [36]. It also reported high usability and likability, specified as being easy to use, demonstrating a preference for the AR application over regular patient education methods, or expressing a desire to use the application in the future [34,36].

In a study by Libaw et al. [37], patients and parents were satisfied with their experience and described less patient anxiety than in previous inductions. AR is concluded to be beneficial as an adjunct or alternative to existing pharmacological and behavioral distraction techniques for preoperative anxiety. The long dressing time group showed less pain than the control group in a study by Gasteratos et al. [33]. That study also reported that the respiratory and pulse rates changed over time, deviating from the trend of the control group, albeit without reaching statistical significance. Furthermore, they reported less pain and negative emotions and reduced parental pain assessment scores [43].

Five studies extracted from Karami et al. [32] focused on training and intervention by implementing an MR environment for participants with ASD to improve their social, emotional, and cognitive skills. Notably, a significant improvement was found in the frequency and duration of pretend play using the AR system compared to a non-computer-assisted situation [38] and in the attention process and appearance of verbal language [41]. The number of participants with ASD and ADHD-related symptoms decreased in both the high- and low- ADHD symptom groups 24 and 48 h after the session [42]. A study found that the correct assessment rates regarding emotion regulation and recognition skills in all children increased, and the mean difference in performance levels was significant [39]. Furthermore, social and communication skills were also improved, with an average ability level in communication significantly improving during and after treatment compared to before intervention in one study [40].

## 4. Discussion

This study is a literature review that systematically explored the impact of care and education using mixed reality (MR) technologies on health-related outcomes for pediatric patients and their families in clinical settings. The data extraction was conducted in two stages, following the PICOS (Population, Intervention, Comparison, Outcome, Study Design) approach. In the first stage, we identified four systematic reviews that included MR interventions for children and their families in clinical or hospital-based settings. These reviews were carefully selected based on their relevance to the pediatric population and were subjected to a final data extraction process. It is important to note that while some of these reviews covered broader populations, only the data specifically relevant to pediatric patients and their families were extracted and analyzed in this study. In the second stage, the four selected reviews were treated as primary studies. From these, we extracted 10 key studies, each detailing specific interventions and their measured effects on health-related outcomes. These 10 studies were then thoroughly analyzed and summarized, focusing on the following eight characteristics: country of origin, study design, characteristics of the study population, primary clinical setting, type of MR device used, nature of the intervention, variables measured, and significant effects observed in the outcome variables. This structured and rigorous approach ensured that the review remained focused on the pediatric population while providing a comprehensive overview of how MR technologies impact health-related outcomes in pediatric clinical settings.

### 4.1. Study Design and Methodological Considerations

The methodological diversity among the studies examined highlights several critical issues in the evaluation of MR technologies. Controlled trials, although essential for establishing causal relationships between interventions and outcomes, often face challenges related to ambiguous randomization processes [34,36,43]. Despite these challenges, controlled trials remain a cornerstone for comparing MR interventions against traditional methods, ensuring the reliability and validity of the findings [46,47,48]. The need for rigorous meta-analyses is evident to further assess the effectiveness of MR approaches in enhancing learning and therapeutic outcomes [46].

### 4.2. Intervention Characteristics and Technological Implementation

MR interventions, particularly those focused on young patients, are constrained by ethical considerations and the vulnerability of the population, limiting the feasibility of randomized controlled trials (RCTs) [49]. For instance, Libaw and Sinskey’s case report on the use of AR as a distraction technique during anesthesia underscores the tailored approach needed for different patient populations [37]. Studies targeting individuals with ASD often employed single-subject experimental designs, a method well-suited to the individualized needs of this population [50]. These studies demonstrate how MR interventions can be adapted to meet the specific requirements of various clinical contexts [38,39,40,41,42].

### 4.3. Population Characteristics and Clinical Settings

The reviewed studies predominantly involved pediatric populations with specific conditions such as chronic diseases, burns, or developmental disabilities. Notably, some studies also included young adults with ASD [42]. The recruitment strategies in these studies reflected the challenges of securing participation from a limited pool of eligible patients, a common issue in pediatric clinical research. The focus on MR’s application in these settings highlights its emerging role in complementing traditional educational and therapeutic techniques, especially in populations without cognitive impairments [34,35,39].

### 4.4. Functional Outcomes and Practical Implications

The MR devices utilized in these interventions were primarily screen-based, requiring hand-held operation. While tablet PCs were most commonly used, AR headsets and smart glasses were also employed, albeit less frequently [37,38,42]. The development frameworks such as Vuforia, ARKit, and ALVAR played a pivotal role in the effectiveness of these interventions by supporting interactive content [34,35,36,37,38,39,40,41,42,43,44,45,46,51,52]. Despite some reports of stability and precision issues in AR applications, the findings suggest significant advancements in the technology, contributing to improved user experiences [53].

### 4.5. Intervention Settings, Technological Implementation, and Functional Outcomes

The settings of the MR interventions varied widely, with a significant focus on non-hospital scenarios, particularly in studies targeting children with ASD. The choice of MR devices, whether tablet PCs, monitor-based setups, or AR headsets, reflects the diverse needs of these interventions. The frequent use of SDKs like Vuforia for content generation in Unity indicates a preference for platforms that offer dynamic and engaging experiences, which are crucial for achieving therapeutic and educational outcomes. The primary outcome variables generally focused on the effectiveness of MR interventions in reducing anxiety, enhancing knowledge acquisition, and improving participant engagement and satisfaction [36,38,39,40,41,42,43]. These outcomes underscore the need for further research to optimize MR interventions in pediatric care [54,55].

### 4.6. Interrelationships between Functional Outcomes

The success of MR interventions is significantly influenced by the interplay between informative, affective, and utilitarian functions. The concept of “flow”, characterized by deep engagement and enjoyment, is particularly relevant in MR applications, where it can enhance both the user experience and the overall effectiveness of the intervention [56,57]. Future research should explore these interrelationships in greater depth, considering how factors like attention, involvement, and interactivity can be leveraged to optimize the design of MR interventions.

### 4.7. Family Involvement, Care Continuity, and Future Research Directions

This study revealed that MR interventions have a significant impact not only on pediatric patients but also on their parents or caregivers. The involvement of parents in therapeutic experiences is crucial, as it can enhance the overall effectiveness of the interventions. When MR interventions are perceived as beneficial by both the patient and their parents, the outcomes are likely to be more positive. MR environments, which allow multiple users to participate and interact, foster a collaborative approach to care and education. This collaborative aspect is particularly beneficial for ensuring continuity of care and education beyond the clinical setting, extending the benefits of the intervention into the home environment. Designing MR interventions that encourage family involvement is essential for maximizing their effectiveness, and future research should investigate how these interventions can sustain care and educational effects over time.

### 4.8. Limitations and Future Research Directions

However, this study had several limitations. The number and quality of the extracted studies may not be sufficient to provide a comprehensive summary and generalization of the findings. Future research should focus on expanding the collection of studies, particularly those with quantitative research designs and meta-analyses, to strengthen the evidence base. The heterogeneity of the studies, with their varied clinical settings and MR applications, also poses challenges in drawing definitive conclusions. Therefore, future studies should aim to develop more controlled study designs that specifically target the pediatric population, with detailed analyses categorized by main aims, subject groups, and clinical environments. Additionally, there is a need for more research to explore the interrelationships between the informative, affective, and utilitarian functions of MR interventions. Understanding these relationships will be crucial for optimizing the design and implementation of MR technologies in pediatric care and education, ensuring that they effectively meet the needs of both patients and their families.

## 5. Conclusions

This literature review examined the impact of MR-based interventions on patient and parental care and education within clinical settings of pediatric departments. Ten studies were extracted from four systematic reviews that demonstrated the effectiveness of MR interventions in the care and education of various diseases. The included studies were classified into eight attributes, and the outcomes were further discussed in terms of informative, definitive, and practical functions. Furthermore, the analysis showed that MR-based pediatric interventions generally provided positive emotional experiences for children and parents, positively impacting learning and treatment outcomes. Therefore, this study provides insight into the key factors explored in the existing literature when developing MR-based intervention programs for pediatric patients and their parents and suggests additional considerations for future program development.

## Figures and Tables

**Table 1:** Research question of the review defined according to the PICOS approach.

P	Patients/Families	Pediatric Patients and/or their Families in Hospitals, Medical Centers (Clinical Settings)
I	Intervention	Use of augmented reality and/or mixed reality technologies in clinical settings
C	Comparison	NA
O	Outcome	Impact on patient health and family-related outcomes
S	Study design	Systematic reviews

PICOS, patient/population, intervention, comparison, and outcomes; NA, not applicable.

**Table 2:** Research question of the review defined according to the PICOS approach.

Database	Query	Results
Scopus	TITLE-ABS-KEY ((“Augmented reality” OR “Mixed reality”) AND (clinical OR medical OR hospital) AND (education OR program OR intervention OR trial) AND (patient OR family OR parent)) AND (LIMIT-TO (PUBYEAR, 2022) OR LIMIT-TO (PUBYEAR, 2021) OR LIMIT-TO (PUBYEAR, 2020) OR LIMIT-TO (PUBYEAR, 2019) OR LIMIT-TO (PUBYEAR, 2018) OR LIMIT-TO (PUBYEAR, >2012) AND (LIMIT-TO (DOCTYPE, “re”))	109
Web of Science	(((TS = (“Augmented reality” OR “Mixed reality”)) AND TS = (clinical OR medical OR hospital)) AND TS = (education OR program OR intervention OR trial)) AND TS = (patient OR family OR parent) AND YEAR PUBLISHED: (>2012) AND DOCUMENT TYPE: Review Article	76

PICOS, patient/population, intervention, comparison and outcomes.

**Table 3:** Characteristics of the included reviews.

Author (Year)	Technologies Used for the Intervention	Main Users (Target Population)	No. of Papers per Review	Method of Analysis	Study Settings	Implications
Urlings et al. (2022) [30]	Augmented reality (AR)	Patients with chronic disease (e.g., prostate cancer, diabetes mellitus, multiple sclerosis, and epilepsy)	10	Qualitative meta- synthesis	Patient education	AR in patient education is limited; therefore, more high-quality studies are needed.
Alqudimat et al. (2021) [31]	Virtual (VR) and augmented reality (AR)	Pediatric patients (e.g., perioperative anxiety/acute/chronic pain)	14	Narrative synthesis	Perioperative settings (operating room and recovery area)	VR intervention is effective and safe. There is only one case report about AR for preoperative anxiety; therefore, more high-quality studies are needed.
Karami et al. (2021) [32]	Virtual (VR) and augmented reality (AR)	Patients with autism spectrum disorder	33	Quantitative synthesis	Clinical settings	The strongest effect was found for daily living skills. Five AR-based interventions showed efficacy. VR-based interventions in clinical settings are highly encouraged, while more high-quality trials are needed.
Gasteratos et al. (2022) [33]	Virtual (VR) and augmented reality (AR)	Burn survivors	58	Qualitative meta- synthesis	Outpatient department/clinical settings (e.g., burn care centers)	Six clinical trials based on VR or AR as nonpharmacologic interventions showed significant pain reduction during wound care and dressing procedures (e.g., rehabilitation, parental/provider satisfaction).

**Table 4:** Extracted elements with corresponding interventions and outcomes.

Country	Study Design	Population Characteristics	Sector (Primary Clinical Setting)	MR Device	Intervention	Variable	Significant Effect (Outcome Variables)	Primary Study	Reviewed Source
USA	Randomized Controlled Trial	Children (n = 91, mean age = 9.5, age range = 7–13 years)	Outpatient facilities	iPad	AR iPad program with a printed storybook (overlay of 3D graphics, Avatar “Remy” and sound)	(1) Patient knowledge (understanding of clinical research) (2) Perception of information delivery (easy to use)	(1) Increase (2-1) Easy to use in parents: 85.0% (2-2) Easy to use in children: 71.2%	[34]	[30]
UK	Mixed methods design	Children (n = 81, female n = 60, mean age = 10.4, age range = 8–14 years)	Outpatient and inpatient department	iPad	Xploro^®^ (Corporation Pop, Manchester, UK) is a DTx platform including an avatar, chatbot, gameplay about health themes, information on a procedure, and coping strategies	(1) Perceived knowledge (2) Anxiety in children (3) Procedural involvement (4) Procedural satisfaction (5) Qualitative interviews about experiences	(1-1) Increased before the intervention (1-2) Significant group x time interaction in favor of the intervention group for the knowledge (2-1) Decreased before the procedure in children (2-2) Decreased before the procedure in parents (3) Increased (5) 80% more aware of how much they ate; 72.5% easy to use; enjoyed, fun, and easy to use; positive hospital experience	[35]	[31]
Spain	NA (there were pre- and post-tests)	Children with diabetes mellitus (n = 70, female n = 41, mean age = 9.2, age range = 5–14 years)	Children attended a conference for patients with diabetes and relatives in 2016	Android device	AR games to support therapeutic education in diabetes (to learn the carbohydrate content of foods)	(1) Pre and post-knowledge (2) Satisfaction (3) Usability	(1) Significant (2) Very high (3) Very high	[36]	[30]
USA	Case report	Boys (n = 3, mean age = 8.7, age range = 8–10 years)	Operating room (during induction of general anesthesia)	Mira (Mira Labs, Inc., Los Angeles, CA, USA) AR headset and iPhone 7	AR software was used as a distraction technique during induction by featuring Jenny the Robot to help patients take deep breaths	NA	Patients and parents described less preoperative anxiety than in previous inductions.	[37]	[31]
UK	Within-subject experiment	Children with autism spectrum disorder or Asperger’s syndrome (n = 12, female n = 2, mean age = 6.8, age range = 4–7 years)	NA	AR objects (three foam blocks and a cardboard box with markers attached), 24-inch monitor, a Logitech webcam Pro 9000, a mini-Bluetooth keyboard, a table, and play materials	Playing with AR toys in mirror AR display to improve and learn pretend to play and representation of pretense	Play observation scale by video analysis (1) Pretend play frequency (2) Pretend play duration (3) Constructive play frequency (4) Constructive play duration	(1) Increased (2) Increased	[38]	[32]
Taiwan	ABAB withdrawal design (withdrawal or reversal design)	Adolescents with autism spectrum disorder (n = 6, female n = 1, mean age = 11.5, age range = 11–13 years)	A 3 m by 6 m room inside a day-treatment room	Sony Vaio Duo Windows 8 tablet	ARVMS (Augmented Reality-based Video-Modeling with Storybook) with seven sessions to learn the facial expressions and emotions of others in social situations	(1) Correct facial expression recognition rate (2) Performance level improvement (assessed by instructor)	(1-1) Significantly improved in all children (1-2) Significant mean difference in performance level between the baseline and follow-up phases	[39]	[32]
Indonesia	Qualitative research (treatment–effect)	Children with autism (n = 12)	School in Pekalongan region	Android-based gadget	Picture Exchange Communication System) for communication training as a multimedia application built on AR technology	(1) Communication ability score (assessed by a teacher)	(1) Increased	[40]	[32]
Colombia	NA	Children with autism (n = 6, female n = 1, mean age = 6, age range = 5–9 years)	Neurorehabilitation clinic in Bogota	Android-based gadget	AR mobile application as a tool for semantical identification therapies	(1) Attention process (no. of children successfully finished the attention task) (2) Appearance of verbal language	(1) An increase of 14% (2) An increase of 9%	[41]	[32]
USA	NA	Children, adolescents, and young adults with autism spectrum disorder (n = 8, female n = 1, mean age = 15, age range = 11.7–20.5 years) - > high ADHD-related group (ABC-H ≥ 13) and low ADHD-related symptom group (ABC < 13)	NA	Google Glass	Empowered Brain, as a smart glasses-based social communication intervention (maintaining gaze toward faces by AR glasses to improve gaze duration to faces and reduce ADHD symptoms)	(1) ABC-H score (a measure of ADHD-related symptoms)	(1) Decrease in the high and low ADHD symptom groups	[42]	[32]
Australia	Prospective randomized controlled trial	Children with acute burns (n = 42, female n = 13, median age = 9, age range = 3–14)	Outpatient department (dressing changes)	AR device (with a 7-inch LCD screen (300 mm × 200 mm × 50 mm, weighing 1000 g, which was connected to an Intel Pentium Trademark 4 computer)	A child can visualize a 3D character called “Hospital Harry” from multiple angles	(1) Pain scores - Faces, Legs, Activity, Cry, and Consolability score for 3–4-years-old and non-verbalizing children - Faces Pain Scale-Revised for verbalizing 4–8-years-old children - Visual Analog Scale (VAS) for 8–14-years-old children (2) Pulse rates, respiratory rates, and oxygen saturation.	(1-1) Significantly lower mean pain scores in the AR group: for the long dressing time (>30 min), over time (1-2) Significantly lower parental VAS score in the AR group than in the control group	[43]	[33]

ADHD, attention-deficit hyperactivity disorder; ABC, antecedent behavior and consequences; 3D, three-dimensional; AR, augmented reality; MR, mixed reality; NA, not applicable; USA, United States Of America; UK, United Kingdom; LCD, liquid crystal display.
